# Indigenous women’s health and data sovereignty: imagining an Indigenous research methodology using quantitative methods

**DOI:** 10.1177/11771801251392925

**Published:** 2025-12-15

**Authors:** Chloe Crosschild, Colleen Varcoe, Helen J. Brown, Annette J. Browne

**Affiliations:** 1 School of Nursing, Faculty of Applied Sciences, The University of British Columbia, Canada; 2 Faculty of Health Sciences, University of Lethbridge, Canada

**Keywords:** Critical Indigenous Feminism, Indigenous Data Sovereignty, Indigenous people, Indigenous research methodology, Indigenous women’s health, quantitative methods, Red Intersectionality, research methods

## Abstract

This article explores Indigenous Research Methodologies (IRM) in the context of Indigenous women’s health, integrating principles of Indigenous Data Sovereignty (IDS) and theoretical ideas from Critical Indigenous Feminism (CIF) and Red Intersectionality (RI). Building on ongoing critiques of dominant Eurocentric health research paradigms, we advocate for IRM to guide quantitative methods that typically exclude Indigenous Peoples and perspectives, perpetuating colonial practices. Incorporating ideas underpinning notions of RI into quantitative studies centers analyses of how colonialism and racism intersect with gender in research with Indigenous women. The authors also call for IDS to ensure Indigenous women have authority over health data and highlight the importance of relational accountability and ethics in research involving Indigenous communities.

## Introduction

Health is defined in current health care systems in ways rooted in dominant Eurocentric paradigms that often erase or dismiss Indigenous Peoples’ understandings of wellness—worldviews that are holistic, relational, and grounded in land, community, spirit, and kinship (Bastien, 2004; Smith, 2021). Given ongoing health inequities for Indigenous women in Canada and globally (Bacciaglia et al., 2023), research must prioritize relational accountability with Indigenous women and support reciprocal capacity building (Varcoe et al., 2011) between Indigenous women and health care institutions. For Indigenous women, health inequities are shaped less by individual behavior than by structural determinants such as racism, colonialism, and land dispossession (Allan & Smylie, 2022; Boyer, 2017; Lansbury & King, 2023; Lawford et al., 2019). Yet dominant narratives continue to focus on individual risk, reinforcing deficit-based frameworks that obscure the root causes of inequity (Hurley, 2024).

These deficit-based approaches are not just incomplete—they perpetuate epistemic racism, or the privileging of western knowledge systems that marginalize Indigenous ethics, methods, and lived experience (Sinclaire et al., 2023). When Indigenous women’s voices and knowledge systems are excluded from the design and interpretation of health data, they become objects of inquiry rather than agents of knowledge. There is an urgent need for research that refrains from pathologizing Indigenous women and instead centers their strengths, agency, and relational responsibilities (Cook, 2016; Sherwood et al., 2015; D. Wilson et al., 2020).

Quantitative research with Indigenous Peoples continues to lack analyses grounded in Indigenous worldviews (Walter & Suina, 2019). Indigenous quantitative spaces—as forms of epistemic resistance and resurgence—require critical engagement with the assumptions of quantitative methods and must be rooted in relational accountability and Indigenous governance (Smylie et al., 2024; Walter & Andersen, 2013). Indigenous Research Methodologies (IRM) can guide this work through frameworks that reflect Indigenous women’s perspectives, sovereignty, and data priorities.

Broad in scope and methods, IRM are not simply an alternative to colonial research paradigms; they are grounded in Indigenous epistemologies and traditions of inquiry that predate and challenge western scientific frameworks. The fundamental tenets of IRM center Indigenous knowledges, traditions, values, and relational ways of being, structuring research that is reciprocal, accountable, and embedded in relationship with Indigenous Peoples, communities, and land (Kovach, 2021; Smith, 2021; S. Wilson, 2008). When activated through a critical and relational standpoint, IRM can guide the uptake of quantitative methods in ways that support Indigenous Data Sovereignty (IDS) and reflect the priorities of Indigenous women in health research.

Indigenous researchers have often been drawn to qualitative methods because these approaches “fit more squarely with Indigenous agendas and community interests than quantitative methods” (Walter & Andersen, 2013, p. 65). Qualitative methods such as interviews, story work, and talking circles often allow for relational accountability, cultural grounding, and inclusion of diverse worldviews in research. These methods are typically flexible and open-ended, allowing Indigenous participants to guide the inquiry. In contrast, some Indigenous scholars argue that quantitative methods are intrinsically tied to positivist and post-positivist paradigms and Eurocentric worldviews (Chilisa, 2011; Kovach, 2021; Smith, 2021), which rest on the assumption that knowledge is objective, measurable, and value-neutral. These epistemological assumptions often conflict with Indigenous perspectives, which understand knowledge as contextual, relational, and shaped by experience, spirituality, and place.

However, the framing of quantitative methods as indivisible from Eurocentric worldviews is not inevitable and Indigenous knowledges can be used to inform and operationalize quantitative analyses (Walter & Suina, 2019). As S. Wilson (2008) notes, it is not the method in isolation that should be held up for scrutiny, but the underlying philosophical and epistemological assumptions, and methodologies in which methods are taken up that determine whether the research is relational and ethical. Quantitative methods can be meaningfully adapted within Indigenous paradigms when guided by Indigenous worldviews, community priorities, and culturally specific forms of validation and interpretation. Without this grounding, quantitative methods risk re-inscribing colonial logics of classification, measurement, and control. Indeed, as discussed later, arguing against the use of quantitative methods without critically examining epistemological assumptions risks rendering their important distinctions invisible.

While much work has been done in creating space for Indigenous worldviews and IRM within qualitative research paradigms, similar efforts have been limited in quantitative research. This is partly due to the epistemological assumptions that often underpin conventional quantitative research—namely, objectivity, generalizability, and separation between researcher and participant. These assumptions conflict with Indigenous paradigms, where knowledge is relational, situated, and shaped by responsibilities to community, land, and spirit. As a result, quantitative health data about Indigenous Peoples is nearly absent of Indigenous voices and knowledges, thereby leaving colonial research practices intact.

However, there are important exceptions that demonstrate how quantitative research can uphold Indigenous governance and self-determination. The work of the First Nations Information Governance Centre (FNIGC), through its advancement of the OCAP© principles (Ownership, Control, Access, and Possession), has established foundational frameworks for Indigenous governance (Konczi & Bill, 2024). Similarly, the International Indigenous Data Sovereignty Interest Group has promoted the CARE Principles (Collective Benefit, Authority to Control, Responsibility, and Ethics) as a global standard for ethical engagement with Indigenous data (Carroll et al., 2023). The *Our Health Counts* initiative (Smylie et al., 2024) offers a compelling model of community-driven, culturally grounded quantitative research that centers First Nations, Inuit, Metis data sovereignty. Grounded in Indigenous principles—such as prioritizing good relationships, viewing research as a gift exchange, and using research as a vehicle for Indigenous community resurgence—this approach illustrates how deliberate, community-centered strategies can challenge colonial frameworks and how quantitative methods can be meaningfully aligned with IRM and IDS to disrupt colonial frameworks and advance Indigenous well-being.

As Walter and Suina (2019) argue, “[a]n Indigenous absence from the field of Indigenous data and quantitative analysis . . . risks absence of Indigenous participation in the framing of the policy directions that flow from those data” (p. 234). This is especially alarming for Indigenous women, whose health needs and lived experiences have long been marginalized through damaged-centered, deficit-based, gender-blind, and pathologizing research frameworks. Indigenous women have been excluded from shaping data collection and interpretation and targeted by systems that use data to justify surveillance, control, and intervention. At stake is both the content of the data, and who defines what counts as health, wellness, and risk.

These dynamics are deeply gendered. For example, Indigenous women are often framed primarily through their reproductive roles, while systemic and intergenerational forms of gendered colonial violence are rendered invisible in policy and research (Anderson, 2020; Loppie & Kent, 2019). Indigenous women’s health is important beyond maternal child health, though their well-being as mothers is especially critical, given their long-standing targeting by colonial systems and violence. Strengthening Indigenous women’s health—and particularly maternal and perinatal health—necessitates not only better data, but data governed by Indigenous women themselves and grounded in relational, culturally specific understandings of wellness (Hurley, 2024). IDS, as it pertains to quantitative methods, is, therefore, critical for reclaiming authority over how Indigenous women’s health is defined, measured, and addressed in both research and policy (Walter et al., 2021). IDS is about creating transformative ways of engaging with Indigenous people’s health, by asking, “What data are needed to meet the needs, priorities, and aspirations of Indigenous peoples?” (Walter & Carroll, 2021, p. 15).

In this article, we outline how Indigenous worldviews and IRM can guide the uptake of quantitative methods in ways that enhance IDS for Indigenous women in the context of maternal child health. In this article, Critical Indigenous Feminism (CIF), specifically Red Intersectionality (RI), is used to inform understanding of Indigenous worldviews. CIF and RI foreground Indigenous women’s resistance to colonial patriarchy, as well as the intersection of gender, racism, and settler colonialism. CIF and RI guide health research to center the lived experiences of Indigenous women, challenge gender-blind approaches, and engage with Indigenous sovereignty through gendered lenses. Extending beyond conventional dichotomies this article advocates for greater engagement with Indigenous worldviews to advance IDS.

## Background and context

In the Canadian context, *Indigeneity* refers to the distinct cultural, social, political, and historical identities and experiences of Indigenous Peoples (Kovach, 2021). This includes unique languages, traditions, and worldviews (Kovach, 2021); enduring connection to ancestral lands and historical experiences (Bello-Bravo, 2019); rights to self-determination and political sovereignty (A. Simpson, 2014); the emphasis on community and relational ways of knowing (L. B. Simpson, 2011; Starblanket, 2018b); and resilience and resistance to colonialism (Coulthard, 2014; L. B. Simpson, 2017; Smith, 2021). The academy has been slow to engage with Indigeneity, and when it has, it has frequently homogenized or obscured the diverse identities and experiences among Indigenous Peoples, particularly marginalizing the intersectional dimensions of gender, ability, sexual orientation, and the voices of women (Smylie et al., 2024). This article takes up RI (Clark, 2016) to deepen this analysis, extending beyond conventional notions of intersectionality by foregrounding Indigenous women’s experiences of colonialism, gendered violence, and dispossession. RI emphasizes Indigenous sovereignty and relational accountability while addressing the intersecting structures that impact Indigenous women’s health, including racism, heteropatriarchy, and state control.

Dominant framings of Indigeneity often privilege individuals in positions of power within “the Indigenous community”, to the detriment of those whose voices and experiences are excluded. For example, the Indian Act (1876/1985), a federal law applied to First Nations Peoples in Canada, continues to shape governance, resource access, and everyday decision-making through Chief and Council systems. Although often framed as community-based, these structures are colonial impositions regulating Indigenous life (Starblanket, 2018a). The Indian Act has also disenfranchised First Nations women and normalized patriarchal governance (Boyer, 2017; A. Simpson, 2014). It privileges Indigenous men through Eurocentric systems that contradict traditional Indigenous governance models, marginalizing those who do not fit this imposed structure. These gendered impacts are not confined to political systems—they also shape how research is designed, negotiated, and interpreted. Researchers must critically consider how “the Indigenous community” is conceptualized and avoid relying solely on nationalist or state-sanctioned political identities (Starblanket, 2018a, p. 10). Indigenous women scholars have long called for research that acknowledges these structural power differentials, addresses colonial histories, and prioritizes relationships, accountability, and community-driven knowledge production (Absolon, 2022; Anderson, 2016; Clark, 2016).

Western research continues to be used as a tool to enforce and maintain colonial processes that portray Indigenous Peoples as inferior to their settler counterparts (Adcock et al., 2019). These trends are continuous with past practices—such as early anthropological studies that perpetuated notions of Indigenous Peoples as “Other”, drawing on primitivist discourses and the “ethnographic gaze” to collect, classify and represent Indigenous cultures through a colonial lens (Smith, 2021, p. 76). In the Canadian context, for example, an ethnography focused on Blackfoot people (Kidd, 1986) described them as a “primitive culture [that] has been so terribly shattered by the impact of civilization” (p. 1). When describing Blackfoot women, the author writes that “Indian women appear to suffer less pain in childbirth, than women in civilized countries” (Kidd, 1986, p. 29). These statements reinforce deeply entrenched stereotypes—such as the *stoic Indigenous woman* or the *hyperfertile but neglectful mother*—which have long been used to dehumanize Indigenous women and justify their exclusion or over-surveillance in health care and child welfare systems (Harding, 2018).

These colonial narratives are not relics of the past—they continue to shape post-positivist health research today. For example, Sheppard et al. (2017) analyzed national birth and death records among Indigenous Peoples in Canada, linking sudden infant death syndrome (SIDS) rates with maternal behaviors and environmental conditions such as overcrowding, smoking, and infant sleep practices; however, there was no analysis of the impacts of colonialism as a precursor to these factors. While identifying individual-level risk, the authors did not situate these conditions within broader histories of displacement, the forced confinement of First Nations peoples to under-resourced reserves, or the chronic underfunding of Indigenous housing and health systems (Lyeo et al., 2024). Recent evidence underscores the importance of expanding the scope of social determinants of health to encompass colonialism as a critical factor (Greenwood et al., 2018). The enduring effects of colonialism—including displacement, cultural disruption, and systemic discrimination—significantly influence health outcomes, particularly among Indigenous populations. These influences manifest in disparities in health care access, elevated rates of chronic diseases, and overall poorer health metrics (Manitowabi, 2025). Recognizing colonialism as a key social determinant of health necessitates systemic changes and the implementation of culturally grounded health interventions to address profound and persistent inequities.

While Sheppard et al. (2017) underscored the potential harms of overcrowded living environments, their analysis notably did not explicitly link such conditions to colonial policies—such as the confinement of Indigenous people to reserves of land often insufficient to survival—and lacked the perspectives of Indigenous women in both the interpretation of the data and the formulation of recommendations. They suggested that the high rates of infant mortality due to SIDS necessitate targeted state interventions in First Nations and Inuit communities, including improved health literacy, access to prenatal care, local birthing opportunities, and postpartum support (Sheppard et al., 2017). While these recommendations may promote greater access to resources, they tend to obscure the structural and colonial root causes of health disparities and instead reinforce individualistic and behavioral framings of risk (Varcoe et al., 2013).

The omission of Indigenous women’s perspectives in data interpretation and policy risks reinforcing colonial frameworks. This renders Indigenous women invisible as knowledge holders and places implicit blame and shame on them as mothers—perpetuating the harmful stereotype of the *incompetent* or *at-risk* Indigenous mother (Harding, 2018). Such narratives are not just discrediting; they are harmful and actively contribute to adverse health outcomes, child welfare surveillance, and over-apprehension, often through decision-making tools that embed racial bias in seemingly neutral assessments (Allan & Smylie, 2022; Denison et al., 2014). As Kukutai and Cormack (2021) argue, this is a form of “data colonialism” (p. 24), where settler epistemologies define what counts as knowledge. Including Indigenous perspectives in data governance and interpretation is not only an equity issue—it is essential for disrupting harm and enabling culturally grounded, community-led health interventions.

Data colonialism fuels colonizing images and discourses that carry ideological power. These narratives create harmful assumptions about Indigenous women, producing stereotypes that reduce their identities to discrediting traits (Fiske & Browne, 2006). For example, Tait (2000) notes how depictions of the “drunken pregnant” or the “violent fetal alcohol affected youth” portray Indigenous women as morally deficient and incapable of self-governance (p. 95). These narratives are reinforced through systemic determinants—racism, land dispossession, and settler colonialism—which undermine Indigenous women’s access to culturally safe, equitable care. Research illuminating health status inequities, such as adverse birth and perinatal health outcomes, cannot and should not be attributed to lifestyle, behaviors or cultural factors, but understood as embodied manifestations of complex socio-historical, political, and economic determinants of health (Browne, 2017). These determinants include poverty, housing insecurity, colonial governance, jurisdictional fragmentation, and the erosion of relational systems of care. Despite this, Indigenous women’s voices are frequently relegated to the margins in health research due to limited analysis of how gender and colonialism intersect.

Promoting IDS requires more than citation—it requires material changes in how data are governed, interpreted, and used (Rhodes et al., 2024). In health research, this includes ensuring that Indigenous Peoples—not state institutions—retain control over data collection, storage, analysis, and sharing. For Indigenous women, IDS also involves reclaiming authority over knowledge systems historically marginalized or silenced. Elders and Knowledge Keepers can guide the definition of health indicators, data interpretation, and culturally meaningful care practices. This shifts data practices from extractive objectivity to relational accountability, grounded in long-term, ethical relationships with participants.

Reclaiming Indigenous women’s roles means re-centering gendered teachings, ceremonial knowledge, and community protocols in how research is conceived and shared. Indigenous knowledge systems emphasize roles that are intergenerational, reciprocal, and embedded in place. IDS is not only about technical infrastructure or policy—it is about upholding Indigenous women’s responsibilities to family, nation, and land through culturally and politically grounded data practices. Aligning research with Indigenous governance and Indigenous feminism makes IDS both a means of protecting data and advancing resurgence.

## Indigenous research methodologies

IRM are grounded in the knowledge systems, values, and relational ethics of Indigenous Peoples. While they vary across nations, IRM share foundational principles such as respect, reciprocity, responsibility, and relationality (Absolon, 2022; Chilisa, 2011; Gaudet, 2019; Kovach, 2021; S. Wilson, 2008). These methodologies are not simply alternative techniques—they are rooted in Indigenous worldviews that position knowledge as relational, contextual, and living. Knowledge is not extracted but shared through relationships with people, land, ancestors, and spirit.

All researchers are influenced by ontological and epistemological assumptions, whether acknowledged or not. These assumptions shape how research questions are formed, how knowledge is interpreted, and how IRM are enacted. IRM prioritize research processes that are accountable to Indigenous communities and grounded in cultural protocols. Methods such as talking circles, ceremony, storywork, and language-based research center Indigenous knowledge and ways of knowing (Absolon, 2022; Archibald, 2008; Kovach, 2021). This often involves working with Elders and knowledge keepers to co-develop research questions and framing concepts of health and well-being through Indigenous languages, guided by ethical commitments to relational accountability and Indigenous governance.

In western literature, methodology is often conflated with methods; while related, they are distinct (Walter & Suina, 2019). Methodology is the theoretical lens or worldview that guides the research approach. Walter and Andersen (2013) describe methodology as “the study of the principles and theories which guide the choice of method”, and “the theoretical lens or worldview through which research is understood, designed, and conducted” (p.40). In this article, methods are understood as research tools used to gather data and are determined by the research question proposed. The cultural, social, and political dimensions of research processes and practices are predominantly encapsulated within the methodology rather than solely within the choice of methods (Walter & Andersen, 2013).

Since the publication of *Decolonizing Methodologies* by Māori scholar Linda Tuhiwai Smith (1999), Indigenous scholars from diverse contexts, including Canada (Archibald et al., 2019; Brown & Strega, 2015; Chilisa, 2011; Hunt, 2018; Kovach, 2021; McGregor et al., 2018; S. Wilson, 2008) have presented their unique engagement with Indigenous research creating a plethora of approaches to IRM. However, their common thread is their prioritization of relational research and ability to maintain and center relationality.

To meaningfully support self-determination, IRM must do more than assert relational values; it must shift power. In Indigenous contexts, being *relational* practices that nurture respectful relationships with land, community, ancestors, and spirit (S. Wilson, 2008)—yet these relationships can be inconsistently enacted or flattened by institutional demands of the broader cosmos (L. B. Simpson, 2017; Wildcat & Voth, 2023). In contrast, *relationality* reflects deeper ontology of interconnectedness and accountability to all beings, including animate and inanimate, human and more than human, spiritual and embodied (Atwood et al., 2024). It frames knowledge as lived and shaped through “responsibilities to kin and Country” (Tynan, 2021, p. 598). Thus, IRM must recognize Indigenous Peoples as knowledge holders, name community members as co-researchers, and ground research in community-defined priorities. Relationality becomes not only an ethical stance, but a political practice that advances Indigenous governance, data sovereignty, and resurgence.

IRM have been pivotal in Indigenous scholarship because they provide pathways that promote research agendas that prioritize the aspirations, needs, and values of Indigenous Peoples and knowledge. As Anishinaabe scholars McGregor et al. (2018) state,

Indigenous research [methodology] offers a clear commitment to recognize and support diversity and nationhood—intellectual self-determination, if you will. Indigenous research explicitly recognizes traditional and contemporary Indigenous knowledge traditions, the value of community leadership and support, and the community’s ownership of knowledge. Indigenous research holds the potential to regenerate and revitalize the life of Indigenous peoples and communities along with the “knowing” that sustains their ongoing vitality. (p. 2)

Indigenous methodologies are not opposite to or a derivative of western tradition. IRM is a distinct epistemological paradigm because it centers Indigenous worldviews, perspectives, values and lived experiences throughout the research processes and practices (Smith, 2021; Walter & Andersen, 2013). Many Indigenous scholars have blended western research methods—such as surveys, statistical analysis, or epidemiological studies—within Indigenous paradigms guided by community needs and cultural protocol (Benoit et al., 2020; Dumont, 2005; Nixon et al., 2017; Smylie et al., 2024; Walter & Suina, 2019). The key is not the method itself, but whether it reflects relational accountability by serving the goals, values, and governance structures of Indigenous communities.

IRM addresses issues of power by making clear the axiology—the ethics or morals that guide the search for knowledge. Grounded in a relational paradigm, Indigenous axiology is founded on the principle of relational accountability—the honest accounting of Indigenous research in relation to the people to whom the research refers and with whom relationships are, therefore, established (S. Wilson, 2001). Relational accountability means honoring relationships with Indigenous Peoples, ancestors, lands, and knowledge systems (S. Wilson, 2008). It involves co-developing questions, sharing interpretation, and ensuring reciprocity throughout. Blending western and Indigenous methods thus ethically supports community priorities and Indigenous sovereignty.

Colonial research practices have long positioned Indigenous Peoples as objects of inquiry, shaping health data and policy through damaged-centered narratives (Drawson et al., 2017). These narratives have materially harmed Indigenous women by justifying surveillance, child apprehension, and systemic neglect (Nixon et al., 2007). IRM rejects these colonial logics by asserting Indigenous governance, knowledge systems, and relational accountability as ethical foundations for research.

A key concept in understanding the persistence of damage-centered narratives is “the Indian Problem” (Smith, 2021), a derogatory term used to justify assimilation, displacement, and containment. Rather than acknowledging colonialism as the source of poverty and marginalization, this framing pathologized Indigenous existence itself (Wolfe, 2006). As Battiste (2007) explains, “[It] is not a problem of [Indigenous] families, but [one]. . . historically produced through systems of colonization. . . underwritten by Western Christianity, defined by white supremacy, and fueled by global capitalism” (p. 150).

In health research, echoes of this framing continue when Indigenous women are pathologized, and when knowledge production reinforces ontological and epistemological orientations that victimize Indigenous women and sustain systems of colonial power and logic (Smith, 2021). IRM offers a relational, accountable, and decolonizing approach that resists the reproduction of “the Indian Problem” narrative and instead centers Indigenous resurgence and self-determined well-being.

## Research methods used in IRM

IRM can guide any research method, including both qualitative and quantitative approaches. However, as argued earlier, research involving Indigenous Peoples has historically lacked the meaningful use of quantitative methods grounded in Indigenous worldviews. While qualitative approaches—such as talking circles and participant observations—have often been favored for their alignment with traditional ways of knowing (S. Wilson, 2008), this methodological pattern reflects deeper structural dynamics.

Indigenous scholars have often gravitated toward qualitative methods in part from a desire to avoid decontextualized, objectifying, exploitative research practices, such as positioning Indigenous Peoples within a deficits-based discourse under the guise of “objectivity” (Walter & Suina, 2019). This trend reflects structural barriers, including institutional ethics, funding structures, and academic norms that privilege western epistemologies and constrain Indigenous-led quantitative spaces (Lopez & Cuasialpud-Canchala, 2025; Walter & Andersen, 2013). The limited use of quantitative methods is not merely methodological, but shaped by these systemic constraints.

Importantly, quantitative methodologies do not necessarily equate with western colonial ideologies or oppressive practices. The argument that proposes qualitative methods align more closely with traditional Indigenous research has been challenged by Palawa and Cochiti Pueblo scholars, Walter and Suina (2019), who claim that “Indigenous Peoples are, and have been, highly numerate in how we understand our worlds. Complex formulas and calculations underpin/ned Indigenous cropping, hunting and navigation to name just a few traditional daily activities” (p. 233). To suggest that Indigenous Peoples are inherently against quantitative analysis perpetuates the colonial narrative that Indigenous knowledges are limited in scope and application, “primitive” and therefore Indigenous people are unable to “count”. Reclaiming quantitative methods is thus not only methodologically important, but also central to Indigenous resurgence and data sovereignty. The continued absence of Indigenous-led quantitative research allows settler hegemony in research to prevail.

## An absence of quantitative methods is problematic

The absence of quantitative methods within IRM is particularly problematic in health care fields, where epidemiological population-based datasets, surveys, and statistical analyses form the evidence base for the development of policy and procedures. Without Indigenous leadership in the quantitative research space, there are serious consequences: evidence that informs health care and social policy is often based on deficit framings, reinforcing colonial narratives about Indigenous Peoples, and justifying discriminatory interventions (Walter & Suina, 2019). This absence impacts Indigenous Peoples at both individual and collective levels, contributing to poor health outcomes, systemic surveillance, and ongoing governance by settler state frameworks (Greenwood et al., 2018).

Currently, numerous quantitative studies continue to pathologize Indigenous women through statistical portrayals that emphasize deficits and risk (Cook, 2016; Sherwood et al., 2015; D. Wilson et al., 2020). This ideologically positions Indigeneity in opposition to whiteness, reinforcing binary logics that uphold colonial dominance and frame Indigenous women as “problems” to be fixed (Smith, 2021). Crucially, the issue lies not with quantitative *methods*, but with colonial epistemologies and *methodologies* used to interpret and assign meaning to the data. Without Indigenous-led approaches that embody relational accountability and Indigenous ways of knowing, quantitative data continue to be operationalized through colonial frameworks, including colonial patriarchy, that perpetuate harm and marginalization.

## CIF and RI

For quantitative analyses to meaningfully address Indigenous women’s health needs, both data colonialism and the gender oppression imposed by colonial patriarchy must be tackled. We argue that CIF and RI provide effective frameworks for this work. CIF and RI reveal how colonial structures simultaneously racialize and gender Indigenous women, and emphasize the need for Indigenous self-determination, relational accountability, and the centering of Indigenous women’s voices within research processes.

The intersecting power structures of gender, racism, and colonialism create profound health inequities for Indigenous women, which require shifts in the way researchers engage with Indigenous women (Lansbury & King, 2023; Loppie & Kent, 2019). Specifically, in health care systems and traditional Indigenous spaces, CIF demands that researchers interrogate the ways in which gender injustice and patriarchy shape the lives of Indigenous women (Suzack, 2015). CIF aligns with IRM’s emphasis on relational accountability and addressing power dynamics. This is achieved by making explicit the axiology (or the ethics and morals) that guides the search for knowledge to promote research agendas that prioritize addressing the intersecting power structures of gender, racism, and colonialism.

At its core, CIF draws attention to the ways in which “settler-colonial states have been and continue to be built out of the dispossession of Indigenous women and the re-ordering of Indigenous relationships/kinship in order to get access to lands” (Anderson, 2020, p. 57). Moreover, Anderson (2020) provides important insights on why Indigenous feminist theorizing emerged in the first place—as a collective effort to “address the particular ways sexism and patriarchy impacted women’s lives in their own communities and nations” (p. 58). Mainstream feminists have often overlooked the distinct experiences of Indigenous women, while dominant Indigenous sovereignty movements have generally neglected to address Indigenous women’s demands for gender equity.

CIF offers a critical lens for rethinking the design and interpretation of quantitative research. Rather than simply documenting disparities, it calls for examining how gendered colonial structures produce them—and for prioritizing Indigenous women’s sovereignty over data about their bodies, families, and nations (Clark, 2016). Quantitative methods must also reflect women’s gendered realities and community priorities. An example of the inter-relatedness of IRM and CIF can be further illuminated through the notion of RI.

RI extends an intersectional approach by addressing the racism that is inherent to colonialism and the material and cultural dispossession that has been accomplished through and on Indigenous women’s bodies (Clark, 2016). RI (Clark, 2016) foregrounds Indigenous resistance, Indigenous sovereignty/nationhood, and anti-colonialism as critical to research and theory. Anti-colonialism primarily focuses on the historical and ongoing struggles against colonial powers and aims to dismantle colonial structures, emphasizing political, social, and economic liberation. In contrast, decolonialism critiques the persistence of colonial impacts in contemporary society, including knowledge systems and cultural practices, advocating for a profound transformation of these structures to address their colonial origins.

Instead of presenting a method or methodology, Clark (2016) uses RI as a framework grounded in Indigenous ontologies—ontologies that are inherently intersectional and challenge dominant western notions of time, age, space, relationship and identity. RI critiques artificial separations of being, arguing that such divisions contradict the Indigenous philosophical principle of holism. By staying true to Indigenous paradigms and recognizing the multifaceted, polyvocal identities of Indigenous women, RI offers an integral shift for IDS that centers Indigeneity and advances decolonial aims.

RI offers a critical corrective in quantitative research, ensuring that design, measurement, and interpretation are informed by Indigenous women’s experiences of colonial, gendered, and racialized structures. RI calls for analysis that rejects deficit narratives and centers Indigenous women’s knowledge, governance, and nationhood—grounding data in accountability rather than performativity, and in sovereignty rather than oppression.

Using IRM to guide collecting quantitative data is essential to advancing Indigenous women’s data sovereignty and health policy, given the powerful influence statistical data holds in governance and health care systems (Walter & Andersen, 2013). An ongoing study, *Iitsim’kaatsita*, led by the first author, exemplifies this approach: Blackfoot women guide the selection, evaluation, and modification of quantitative scales to measure perinatal experiences, while assessing familiarity with and access to traditional cultural and spiritual practices. Grounded in IRM and RI, the study ensures data reflect Indigenous realities and supports health sovereignty, not colonial control.

## Imagining an IRM using quantitative methods

Specific data needs vary across Indigenous Peoples and Nations, but overall there is a collective need for data that meet Indigenous Peoples’ aspirations which include, “data that disrupt deficit narratives, data that are disaggregated, data that reflect the embodied social, political, historical, and cultural realities of Indigenous people’s lives, as Indigenous peoples, and data that address Indigenous nation rebuilding agendas” (Walter & Suina, 2019, p. 236). Utilizing the relational ontological and epistemological underpinnings of an Indigenous worldview and the principles of IRM, that include centering Indigeneity through Indigenous knowledges, tradition, and values, can guide the uptake of quantitative methods.

For many Indigenous women, health is not solely about physical health well-being, but can also be deeply connected to Indigenous ways of life, encompassing spirituality, relationships with human and non-human kin, and a connection to the land (Million, 2021). Data that reflect these conceptualizations of health and well-being are needed. It is not enough to simply make visible the terms that are used to conceptualize Indigenous People’s participation in health research, such as “partnership” and “consultation”. Rather these terms need to be operationalized and upheld in meaningful ways; neglecting to carry through on meaningful relationship and partnership is tokenism. Embodying partnership invites Indigenous women’s leadership over collecting data, which is essential to research involving Indigenous women and IDS. Therefore, for quantitative data to be meaningful, it is essential to include Indigenous women’s input on what they consider important, reflecting concepts tied to their experiences. In this way IRM can more effectively contribute to eradicating colonial processes (Walter & Suina, 2019).

Several initiatives demonstrate the possibilities of Indigenous-led, relational quantitative research. Projects such as *Our Health Counts* (Smylie et al., 2024), the First Nations Regional Health Survey (Dumont, 2005), and *Reclaiming Our Spirits* (Varcoe et al., 2017, 2021) show how quantitative methods can be shaped by Indigenous paradigms—prioritizing Indigenous governance, validation, relational accountability, and cultural specificity within IRM frameworks.

For example, in *Iitsim’kaatsita* Blackfoot women co-designed survey questions to privilege the strengths of traditional Indigenous birthing and childrearing practices and make space for them to indicate their feelings of safety when incorporating traditional, cultural, or ceremonial practices into their perinatal experience. Utilizing IRM can center Indigenous women’s conceptualization of Indigeneity through community-engaged discussions in ways that can prevent sentimental or essentialist views of relationships as inherently positive in Indigenous communities, recognizing that not all relationships are harmonious; some may be fraught or challenging, yet they remain crucial to navigate in sustaining community and kinship networks (Gaudry, 2015; Starblanket, 2018b).

IRM with CIF ensures Indigenous women guide and maintain control of what questions are asked and who gets to ask the questions about health based on their understandings and experiences of health and Indigeneity. In addition, CIF centers analysis on gender, sexuality, racism, Indigeneity, and nation (Arvin et al., 2013). Accordingly, it is important for inquiries focused on Indigenous women’s health to take into consideration the impacts of gendered violence (L. B. Simpson, 2017).

In the study conducted in Canada known as *Reclaiming Our Spirits* (Varcoe et al., 2017, 2021), quantitative data were collected in ways that privileged Indigenous women’s voices. The study evaluated a health promotion intervention for women experiencing violence, developed specifically for Indigenous women. While not explicitly framed as IRM, the researchers followed IRM principles by (a) establishing a steering committee of Indigenous women leaders; (b) drawing on literature by Indigenous scholars; (c) using Cree concepts to guide the teams’ thinking beyond English; (d) receiving guidance from Elders from various Nations; and (e) involving participants in refining survey instruments, approving analyses, naming the study, and acknowledging their contributions. These practices helped create the conditions necessary for conducting research with Indigenous women in this context.

Quantitative methods driven by IRM will (a) be guided by Indigenous perspectives; (b) include, such as advised by RI, understanding the impact of colonialism and racism on Indigenous people’s experiences (in this case, Indigenous women’s experiences as it pertains to gender and as it intersects with colonial patriarchy); and (c) will scrutinize each element of methodology. Again, using the example of the ongoing study, *Iitsim’kaatsita*, Blackfoot Elders, Knowledge Keepers and leaders guided the development of the research questions and the selection and adaptation of survey instruments, such as the Everyday Discrimination Scale (Williams et al., 1997). They subsequently reviewed preliminary statistical analyses and provided guidance on ways to incorporate spirit, through Blackfoot ceremony, into the dissemination of data. Potential participants reviewed and piloted data collection instruments and offered feedback that enhanced the survey questions in meaningful ways, such as including strength-based language, privileging Blackfoot ways of knowing as acceptable, important forms of education in the demographic questions, and questions about advocacy for receiving equitable care. This research is currently in progress and will form the basis for future analyses informed by CIF and IRM.

## Conclusion

This article emphasizes the importance of IRM using quantitative methods to address the health inequities faced by Indigenous women. By centering Indigenous perspectives, IRM offers a framework that respects and prioritizes Indigenous knowledge systems, relational accountability, and sovereignty over data. This approach not only challenges the colonial and gendered biases prevalent in traditional research methods but also empowers Indigenous women to take control of their health narratives and outcomes.

The use of quantitative methods within an IRM framework provides a powerful tool to quantify and address the impacts of colonialism, racism, and patriarchy on Indigenous women’s health. This methodology ensures that research is conducted with Indigenous Peoples, respecting local cultural protocols and knowledge systems. By involving Indigenous communities in every step of the research process, from the development of research questions to the dissemination of findings, IRM fosters a collaborative and respectful research environment.

Moreover, the integration of CIF within IRM highlights the intersectional nature of Indigenous women’s experiences, addressing the compounding effects of gender, racism, and colonialism. This approach ensures that the unique health needs and perspectives of Indigenous women are not only acknowledged but are central to the research agenda.

In conclusion, adopting an IRM framework with quantitative methods is crucial for advancing Indigenous women’s health research. This approach not only disrupts deficit-based narratives but also contributes to the broader goals of IDS and nation-building. By prioritizing Indigenous women’s voices and leadership in health research, action toward more equitable and culturally relevant health outcomes for Indigenous communities can be undertaken.
